# Serum electrolyte abnormalities in cats with chronic inflammatory enteropathy

**DOI:** 10.1111/jvim.17242

**Published:** 2024-11-08

**Authors:** Iona Baker, Romy Heilmann, Ramona Knoll, Berenice Schneider, Yuvani Bandara, Simon Priestnall, Aarti Kathrani

**Affiliations:** ^1^ Royal Veterinary College University of London London AL97TA United Kingdom; ^2^ University of Leipzig Leipzig Sachsen Germany; ^3^ Colorado State University Fort Collins Colorado USA

**Keywords:** diarrhea, feline, intestine, potassium, sodium, vomiting

## Abstract

**Background:**

Limited information is available on electrolyte abnormalities in cats with chronic inflammatory enteropathy (CIE).

**Hypothesis/Objectives:**

Report the prevalence of electrolyte abnormalities in cats with CIE compared to other gastrointestinal disorders, and determine their association with disease and outcome variables in cats with CIE.

**Animals:**

Three hundred twenty‐eight client‐owned cats from 2 referral hospitals: CIE (132), alimentary small cell lymphoma (29), acute gastroenteritis (48), and healthy controls (119).

**Methods:**

Retrospective study comparing serum electrolyte concentrations at time of diagnosis among the 4 groups of cats, and associations with clinical signs, intestinal mucosal fibrosis scores, treatment subclassification and outcome in CIE.

**Results:**

Cats with CIE had lower sodium and higher potassium concentrations and lower sodium: potassium ratios compared with healthy cats (*P* < .001, *P* = .01, and *P* < .001, respectively). Cats with CIE and a duodenal mucosal fibrosis score of 2 had lower sodium and lower total calcium concentrations compared with cats that had a score of 0 (*P* = .02 and *P* = .01). Cats with CIE and a colonic mucosal fibrosis score of 1 had higher potassium concentrations and lower sodium: potassium ratios compared with cats that had a score of 0 (*P* = .03 and *P* = .01). Cats with CIE that died as a result of their disease had higher potassium concentrations and lower sodium: potassium ratios compared to cats that were alive (*P* = .02 and *P* = .01).

**Conclusions and Clinical Importance:**

Electrolyte abnormalities occur with CIE and, in particular, in cats with higher fibrosis scores and worse outcomes. Further research should aim to determine the pathogenesis of these findings and identify novel therapeutic targets for cats with CIE.

**Abbreviations**
CBC: complete blood count
CIE: chronic inflammatory enteropathy
DSH: domestic short hair
EnaC: electrogenic epithelial sodium channels
fPLI: feline pancreatic lipase
GE: gastroenteritis
IBD: inflammatory bowel disease
IHC: immunohistochemistry
PARR: antigen receptor rearrangements
PCR: polymerase chain reaction
SCL: small cell lymphoma
TLI: trypsin like immunoreactivity
TT4: total thyroxine
WSAVA: World Small Animal Veterinary Association

## 1 INTRODUCTION

Chronic enteropathy in cats is defined as a group of disorders resulting in intermittent or persistent gastrointestinal signs of at least 3 weeks' duration, where standard diagnostic investigations, including laboratory tests, fecal analysis, and abdominal imaging, have ruled out all identifiable causes, such as secondary (extra‐gastrointestinal disease), as well as infectious, obstructive, structural or neoplastic gastrointestinal disease.^1^, ^2^, ^3^ For cases of chronic enteropathy in which histopathology of gastrointestinal biopsy specimens shows inflammatory infiltrates, morphological changes or both, the term chronic inflammatory enteropathy (CIE) typically is used.^4^ Chronic inflammatory enteropathy is an idiopathic condition, although the pathogenesis is hypothesized to involve the variable interplay of 4 key components: genetic susceptibility, environmental risk factors, intestinal dysbiosis, and altered gastrointestinal mucosal immune response.^5^ Treatment involves a sequential or combination approach using therapeutic diets and immunosuppressants. However, some cats may not respond to treatment, with 1 study showing 37% of cats with CIE ultimately being euthanized as a consequence of their disease.^6^ Therefore, a better understanding of the pathogenesis of this disease may allow identification of novel therapeutic targets that alone or in combination may render treatment more effective and result in better outcomes for affected cats.

Inflammatory bowel disease (IBD) in people shares some similarities with CIE in cats, with both conditions being chronic and idiopathic with a relapsing course.^7^, ^8^ In people, in comparison with healthy subjects, increased potassium secretion and decreased sodium absorption in the gastrointestinal tract are seen in IBD syndromes, Crohn's disease and ulcerative colitis.^9^ It also has been shown, particularly for ulcerative colitis, that the colon becomes less prone to absorption and more prone to secretion, and thus there is less water and sodium absorption and more potassium secretion.^9^ Therefore, people with IBD are prone to electrolyte abnormalities, particularly hyponatremia, hypokalemia, and hypochloremia.^9^ Indeed, hyponatremia was the most frequent electrolyte abnormality documented in people with IBD and it was attributed primarily to decreased absorption of sodium.^10^ Interestingly, the prevalence of electrolyte abnormalities in dogs with CIE is similar to that of humans with IBD, however the most frequent electrolyte abnormality reported in dogs with CIE was hypokalemia at 19%, although 14% were also hyponatremic.^11^ These findings may reflect differences in the disease process and the compensatory mechanisms between humans with IBD and CIE in dogs. Currently, serum electrolyte abnormalities have not been adequately described in cats with CIE. Determining the frequency and type of electrolyte abnormalities in cats with CIE is of interest, because recently we have shown that for cats with CIE, the presence of colonic fibrosis worsens the disease outcome.^12^ Therefore, because the colon is involved in the absorption and secretion of electrolytes, determining what electrolyte abnormalities are present in cats with CIE and how they are associated with clinical and outcome variables might provide further insight into disease pathogenesis and suggest novel targets for more effective treatment.

We aimed first to compare serum electrolyte concentrations in cats with CIE to cats with acute gastroenteritis (acute GE), alimentary small cell lymphoma (SCL) and healthy cats, and second, to identify associations between serum electrolyte concentrations and clinical signs, intestinal mucosal fibrosis scores, treatment subclassification and outcome in cats with CIE. We hypothesized that electrolyte abnormalities in cats with CIE would be similar to those in dogs with CIE, and that these electrolyte abnormalities would be more severe in cats with higher intestinal mucosal fibrosis scores and worse disease outcomes.

## 2 MATERIALS AND METHODS

### 2.1 Case selection

The medical records at the Royal Veterinary College and University of Leipzig were retrospectively searched for cats diagnosed with CIE, alimentary SCL, and acute GE. Additionally, the medical records were searched for healthy blood donor cats. Inclusion criteria for CIE included a minimum of 3 weeks of persistent or intermittent gastrointestinal signs (vomiting, diarrhea, hyporexia, weight loss) and diagnostic investigations that ruled out known causes, including CBC, serum biochemistry profile including electrolytes, serum cobalamin or folate concentrations or both, serum total thyroxine (TT4) concentration if >8 years of age,^13^ pancreatic function testing (feline pancreatic lipase [fPLI] and trypsin‐like immunoreactivity [TLI]) as indicated by historical findings, physical examination and diagnostic imaging findings, fecal parasitology or empirical deworming or both, *Tritrichomonas foetus* PCR assay for large intestinal diarrhea, transabdominal ultrasonography and histopathologic findings from intestinal biopsy specimens obtained by endoscopy or laparotomy, with or without immunohistochemistry (IHC) and PCR for antigen receptor rearrangements (PARR), consistent with CIE. Exclusion criteria for CIE included cats that had incomplete diagnostic investigations to rule out relevant causes and in which histopathology with or without IHC and PARR of intestinal biopsy specimens yielded a diagnosis of alimentary neoplasia. In total, 132 cats met the criteria for CIE and were included in the study. Inclusion criteria for alimentary SCL included compatible clinical signs and a definitive diagnosis based on intestinal histopathology with or without IHC and PARR by a board‐certified veterinary pathologist (n = 29). Inclusion criteria for acute GE included cats that had <3 weeks of persistent or intermittent gastrointestinal signs in which diagnostic investigations ruled out secondary or extra‐gastrointestinal causes and in which the diagnosis was primary gastrointestinal disease (parvoviral enteritis; n = 42) or a definitive cause was not identified (n = 6). Of the 48 cats with acute GE, 1 that was diagnosed with parvoviral enteritis also had a concurrent obstructing duodenal foreign body. No other cats were diagnosed with a gastrointestinal foreign body. A total of 119 cats used as blood donors comprised the healthy group of cats used in the study. Blood donor cats at both referral hospitals were deemed healthy based on history, physical examination findings, CBC, serum biochemistry and relevant infectious disease testing.

### 2.2 Data collection

Hospital records and results of diagnostic investigations were reviewed for all cats to ensure eligibility for the respective disease categories of CIE, alimentary SCL, acute GE and healthy control. The information recorded consisted of age, sex and neuter status, breed and serum sodium, chloride, corrected chloride, potassium, total calcium, phosphorus, and total magnesium concentrations, as well as sodium: potassium ratio and calcium phosphorus product. Absolute and relative values for all electrolytes were included to standardize the data across the 2 referral hospital laboratories. Relative values were determined as ([measured electrolyte]/[lower reference limit]). Corrected chloride was determined as ([mid‐reference interval sodium/measured sodium] × [measured chloride]).^14^ For each cat diagnosed with CIE, presenting clinical signs (vomiting, diarrhea, decreased appetite, weight loss), World Small Animal Veterinary Association (WSAVA) duodenal and colonic mucosal fibrosis score, treatment subtype (food‐responsive, antimicrobial‐responsive, and immunosuppressant‐responsive), and outcome consisting of death caused by gastrointestinal disease, or death of other causes and alive also were recorded. Because the study exclusively used retrospective case data from hospital records, ethical approval was not required by 1 of the hospital's ethical review boards and for the second hospital, approval was granted by the relevant ethical review board (Royal Veterinary College [URN SR2024‐0045R]).

### 2.3 Statistical analysis

A commercially available computer software package (IBM SPSS Statistics v29, Chicago, IL) was used for all statistical analyses. Histograms and the Shapiro‐Wilk test were used to assess for normality of continuous variables.

A Chi‐squared test was used to compare the proportion of domestic short hair (DSH) and non‐DSH breed and sex and neuter status among the 4 groups of cats. The Kruskal‐Wallis test with pairwise comparisons was used to compare age at diagnosis among the 4 groups of cats.

A Chi‐squared test was used to compare the proportion of different electrolytes that were below, within or above their laboratory reference interval among the 4 groups of cats. The Kruskal‐Wallis test with pairwise comparisons was used to compare absolute and relative serum concentrations of electrolytes among the 4 groups of cats. For cats with CIE, when electrolytes were compared to outcomes with 2 categories, a Mann‐Whitney *U* test was used (vomiting vs no vomiting, diarrhea vs no diarrhea, decreased appetite vs normal appetite, weight loss vs no weight loss, death caused by gastrointestinal disease vs death of other cause and alive), and for outcomes with >2 categories (treatment subclassification and WSAVA duodenal and colonic mucosal fibrosis scores), a Kruskal‐Wallis test followed by pairwise comparisons was used. Significance was defined as *P* < .05 or adjusted *P* < .05 for pairwise comparisons using Bonferroni correction.

## 3 RESULTS

### 3.1 Study cats

Three hundred twenty‐eight client‐owned cats were included from 2 referral hospitals: CIE (132), alimentary SCL (29), acute GE (48), and healthy controls (119). The cats with CIE included in the study consisted of 69 neutered males, 5 intact males, 53 neutered females and 5 intact females. Age ranged from 4 to 206 months, with a median of 102.5 months. Breeds included 84 DSH and 48 non‐DSH. The cats with alimentary SCL consisted of 16 neutered males, 3 intact males and 9 neutered females, with 1 unknown sex and neuter status. Age ranged from 69 to 206 months, with a median of 139 months. Breeds included 21 DSH and 8 non‐DSH. The cats with acute GE consisted of 7 neutered males, 21 intact males, 7 neutered females and 13 intact females. Age ranged from 1 to 139 months with a median of 5 months. Breeds included 27 DSH and 21 non‐DSH. The healthy control cats consisted of 63 neutered males, 8 intact males, 42 neutered females, and 6 intact females. Ages ranged from 9 to 167 months, with a median of 63 months. Breeds included 82 DSH and 37 non‐DSH.

No significant differences in breed (DSH and non‐DSH) were found among the 4 groups (*P* = .36). Sex and neuter status were significantly different among the 4 groups (*P* < .001). Age was not normally distributed (*P* < .05). A significant difference in age was found among the 4 groups (*P* < .001) with pairwise comparisons showing the following significant differences: Acute GE compared to healthy, CIE and alimentary SCL (adjusted *P* < .001, adjusted *P* < .001, and adjusted *P* < .001, respectively), healthy compared to CIE and alimentary SCL (adjusted *P* < .001 and adjusted *P* < .001, respectively), and CIE compared to alimentary SCL (adjusted *P* = .02).

For the CIE group, 81 cats (61%) had vomiting, 57 (43%) had diarrhea, 81 (61%) had decreased appetite and 63 (48%) had weight loss. The median WSAVA duodenal mucosal fibrosis score was 0 (range, 0‐3) and the median WSAVA colonic mucosal fibrosis score was 0 (range, 0‐2). Fifty of 115 cats (43%) were subclassified as food‐responsive, 8 (7%) as antibiotic‐responsive and 57 (50%) as immunosuppressant‐responsive; data regarding treatment subclassification was unavailable for 17 cats. Twenty‐nine of 108 cats (27%) died as a result of their gastrointestinal disease and 79 (73%) were alive or dead of non‐gastrointestinal related causes; data regarding outcome was unavailable for 24 cats.

### 3.2 Serum electrolyte concentrations in cats with CIE, alimentary SCL, acute GE, and healthy controls

All serum electrolyte concentrations were not normally distributed (*P* < .05).

The proportions of cats with CIE, alimentary SCL, acute GE, and healthy controls with serum sodium, potassium, chloride, total calcium and phosphorus concentrations below, within and above the laboratory reference interval are summarized in Tables 1, 2, 3, 4, 5. Chi‐squared tests showed a significant difference in the proportion of serum sodium, potassium, chloride and phosphorus concentrations below, within and above the laboratory reference interval among the 4 groups of cats (*P* < .001, *P* < .001, *P* = .02, and *P* < .001, respectively). No significant difference was found in the proportion of serum total calcium concentrations below, within and above laboratory reference intervals among the 4 groups of cats (*P* = .2).

**TABLE 1:** The number of cats and percentages with serum sodium (Na) concentration below, within or above the laboratory reference interval at the time of presentation for different gastrointestinal diseases (CIE = chronic inflammatory enteropathy, SCL = alimentary small‐cell lymphoma, acute GE = acute gastroenteritis) and healthy controls.

	Disease category			Healthy	Total
	CIE	SCL	Acute GE		
Na					
Low	12 (9.1%)	2 (7.7%)	21 (41.2%)	0 (0%)	35 (10.7%)
Normal	118 (89.4%)	23 (88.5%)	29 (56.9%)	113 (95.0%)	283 (86.3%)
High	2 (1.5%)	1 (3.8%)	1 (1.9%)	6 (5.0%)	10 (3.0%)
Total	132	26	51	119	328

*Note*: Chi‐squared test showed that the proportion of serum sodium concentrations below, within or above the laboratory reference interval differed significantly among the 4 groups of cats (*P* < .001).

**TABLE 2:** The number of cats and percentages with serum potassium (K) concentration below, within or above the laboratory reference interval at the time of presentation for different gastrointestinal diseases (CIE = chronic inflammatory enteropathy, SCL = alimentary small‐cell lymphoma, acute GE = acute gastroenteritis) and healthy controls.

	Disease category			Healthy	Total
	CIE	SCL	Acute GE		
K					
Low	14 (10.6%)	1 (3.8%)	11 (21.6%)	9 (7.6%)	35 (10.7%)
Normal	112 (84.8%)	25 (96.2%)	33 (64.7%)	108 (91.5%)	278 (85.0%)
High	6 (4.5%)	0 (0.0%)	7 (13.7%)	1 (0.8%)	14 (4.3%)
Total	132	26	51	118	327

*Note*: Chi‐squared test showed that the proportion of serum potassium concentrations below, within or above the laboratory reference interval between the 4 groups of cats was significant (*P* < .001).

**TABLE 3:** The number of cats and percentages with serum chloride (Cl) concentration below, within or above the laboratory reference interval at the time of presentation for different gastrointestinal diseases (CIE = chronic inflammatory enteropathy, SCL = alimentary small‐cell lymphoma, acute GE = acute gastroenteritis) and healthy controls.

	Disease category			Healthy	Total
	CIE	SCL	Acute GE		
Cl					
Low	2 (1.6%)	3 (12.0%)	0 (0%)	7 (5.9%)	12 (3.7%)
Normal	99 (77.9%)	19 (76.0%)	36 (70.6%)	96 (80.7%)	250 (77.6%)
High	26 (20.5%)	3 (12.0%)	15 (29.4%)	16 (13.4%)	60 (18.6%)
Total	127	25	51	119	322

*Note*: Chi‐squared test showed that the proportion of serum chloride concentrations below, within or above the laboratory reference interval between the 4 groups of cats was significantly different (*P* = .02).

**FIGURE 1:**
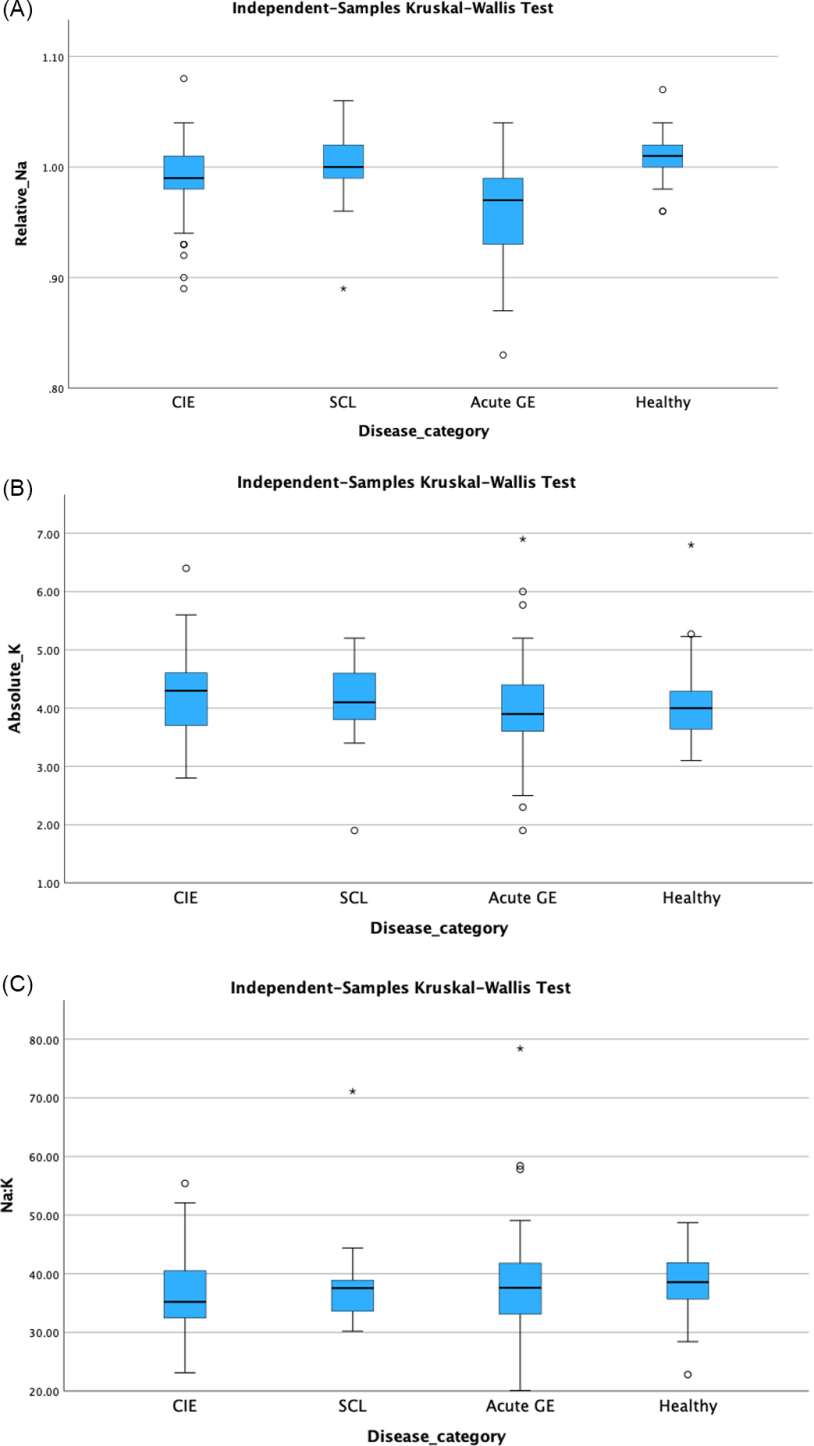
Box and whisker plot showing (A) relative serum sodium concentrations, (B) absolute serum potassium concentrations, and (C) serum sodium: Potassium ratio between cats with different gastrointestinal diseases (CIE = chronic inflammatory enteropathy, SCL = alimentary small‐cell lymphoma, acute GE = acute gastroenteritis) and healthy controls. The box represents the interquartile range, with the bottom and top edges of the box representing the 25th and 75th percentiles, respectively. The vertical line that splits the box in 2 represents the median. The bottom and top whiskers represent the minimum and maximum values, unless there is an outlier. If an outlier is present (represented as a circle or a star if an extreme outlier), the whiskers represent 1.5 times the interquartile range. Cats with CIE had a lower relative serum sodium concentration compared to healthy cats (adjusted *P* < .001), and a higher relative serum sodium concentration compared to cats with acute GE (adjusted *P* < .001). Cats with CIE also had higher absolute and relative serum potassium concentrations, and lower serum sodium: Potassium ratios compared to healthy cats (adjusted *P* = .01, adjusted *P* = .01, and adjusted *P* < .001, respectively).

Cats with CIE had lower absolute and relative serum sodium concentrations compared with healthy cats (adjusted *P* < .001), and higher absolute and relative serum sodium concentrations compared with cats with acute GE (adjusted *P* < .001; Figure 1A). Cats with CIE had higher absolute and relative serum potassium concentrations (adjusted *P* = .01 and *P* = .01; Figure 1B), and lower serum sodium: potassium ratios compared with healthy cats (adjusted *P* < .001; Figure 1C). No other significant differences in serum electrolyte concentrations were observed among the 4 groups of cats (*P* > .05).

**TABLE 4:** The number of cats and percentages with serum total calcium (tCa) concentration below, within or above the laboratory reference interval at the time of presentation for different gastrointestinal diseases (CIE = chronic inflammatory enteropathy, SCL = alimentary small‐cell lymphoma, acute GE = acute gastroenteritis) and healthy.

	Disease category			Healthy	Total
	CIE	SCL	Acute GE		
tCa					
Low	12 (10.5%)	5 (19.2%)	4 (19.0%)	1 (2.0%)	22 (10.4%)
Normal	100 (87.7%)	21 (80.8%)	17 (81.0%)	49 (96.0%)	187 (88.2%)
High	2 (1.7%)	0 (0.0%)	0 (0.0%)	1 (2.0%)	3 (1.4%)
Total	114	26	21	51	212

*Note*: Chi‐squared test showed that the proportion of serum total calcium concentrations below, within or above the laboratory reference interval among the 4 groups of cats was not significant (*P* = .2).

**TABLE 5:** The number of cats and percentages with serum phosphorus (Phos) concentration below, within or above the laboratory reference interval at the time of presentation for different gastrointestinal diseases (CIE = chronic inflammatory enteropathy, SCL = alimentary small‐cell lymphoma, acute GE = acute gastroenteritis) and healthy controls.

	Disease category			Healthy	Total
	CIE	SCL	Acute GE		
Phos					
Low	2 (1.8%)	3 (12.0%)	0 (0.0%)	1 (2.0%)	6 (2.9%)
Normal	107 (96.4%)	21 (84.0%)	12 (63.2%)	49 (98.0%)	189 (92.2%)
High	2 (1.8%)	1 (4.0%)	7 (36.8%)	0 (0.0%)	10 (4.9%)
Total	111	25	19	50	205

*Note*: Chi‐squared test showed that the proportion of serum phosphorus concentrations below, within or above the laboratory reference interval differed significantly among the 4 groups of cats (*P* < .001).

### 3.3 Serum electrolytes in cats with CIE and association with clinical signs, intestinal mucosal fibrosis scores, treatment subclassification and outcome in cats with CIE

#### 3.3.1 Serum electrolyte abnormalities in cats with CIE

Increased serum chloride concentration was the most frequent electrolyte abnormality in cats with CIE, with a frequency of 19.7% (n = 26). The second most common electrolyte abnormality was hypokalemia (10.6%), followed by hyponatremia (9.1%) and total hypocalcemia (9.1%). Hypophosphatemia and hyperphosphatemia were among the least frequent abnormalities (1.5% and 1.5%; Table 6).

**TABLE 6:** The frequencies and percentages of cats with chronic inflammatory enteropathy (CIE) that presented with serum sodium, potassium, chloride, total calcium and phosphorus concentrations below or above the respective reference interval (RI).

	Frequency	Percent
Sodium		
Below RI	12	9.1
Above RI	2	1.5
Potassium		
Below RI	14	10.6
Above RI	6	4.5
Chloride		
Below RI	2	1.5
Above RI	26	19.7
Total calcium		
Below RI	12	9.1
Above RI	2	1.5
Phosphorus		
Below RI	2	1.5
Above RI	2	1.5

#### 3.3.2 Serum electrolytes and clinical signs

**FIGURE 2:**
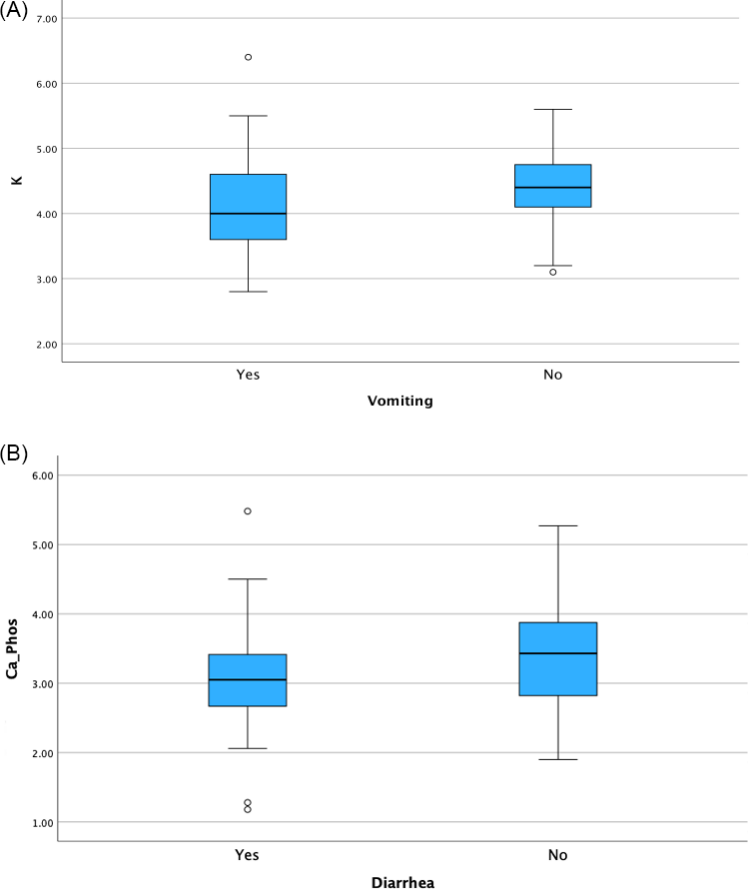
Box and whisker plot showing (A) the absolute serum potassium concentrations of cats with chronic inflammatory enteropathy (CIE) that presented with or without vomiting and (B) the serum calcium × phosphorus of cats with CIE that presented with or without diarrhea. The box represents the interquartile range, with the bottom and top edges of the box representing the 25th and 75th percentiles, respectively. The vertical line that splits the box in 2 represents the median. The bottom and top whiskers represent the minimum and maximum values, unless there is an outlier. If an outlier is present (represented as a circle or a star if an extreme outlier), the whiskers represent 1.5 times the interquartile range. Cats with CIE that had vomiting had lower absolute serum potassium concentrations compared with cats without vomiting (*P* = .03). Cats with CIE that presented with diarrhea had a lower calcium × phosphorus product than those cats without diarrhea (*P* = .04).

Cats with CIE that presented with vomiting had lower absolute and relative serum potassium concentrations compared with cats without vomiting (*P* = .03; Figure 2A). Cats with CIE that presented with diarrhea had lower calcium × phosphorus products than those without diarrhea (*P* = .04; Figure 2B). No significant associations were found between serum electrolyte concentrations and whether or not cats presented with decreased appetite or weight loss (*P* > .07).

#### 3.3.3 Serum electrolytes and duodenal and colonic mucosal fibrosis

**FIGURE 3:**
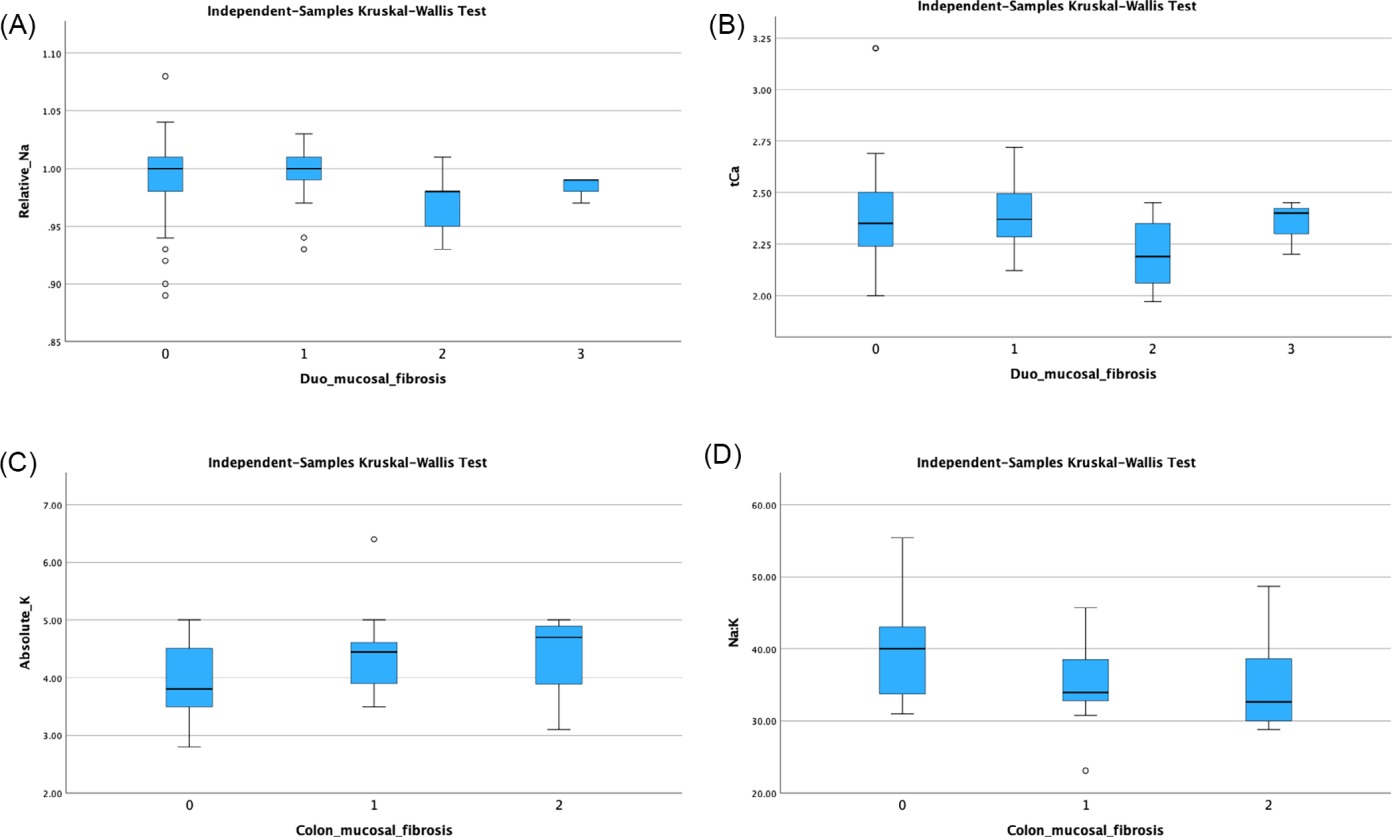
Box and whisker plot of (A) relative serum sodium concentrations and (B) absolute serum total calcium concentrations in cats with chronic inflammatory enteropathy (CIE) and different duodenal mucosal fibrosis scores and (C) serum absolute potassium concentration and (D) sodium: Potassium ratio in cats with CIE and different colonic mucosal fibrosis scores. The box represents the interquartile range, with the bottom and top edges of the box representing the 25th and 75th percentiles, respectively. The vertical line that splits the box in 2 represents the median. The bottom and top whiskers represent the minimum and maximum values, unless there is an outlier. If an outlier is present (represented as a circle or a star if an extreme outlier), the whiskers represent 1.5 times the interquartile range. Cats with CIE and a duodenal mucosal fibrosis score of 2 had lower relative serum sodium concentrations compared to cats with a score of 0 (adjusted *P* = .02). Cats with CIE and a duodenal mucosal fibrosis score of 2 had lower total calcium concentrations compared to cats with a score of 1 and 0 (adjusted *P* = .02 and adjusted *P* = .01, respectively). Cats with CIE and a colonic mucosal fibrosis score of 1 had higher absolute serum potassium concentrations and lower sodium: Potassium compared to cats with a score of 0 (adjusted *P* = .03 and adjusted *P* < .01, respectively).

Cats with CIE and a duodenal mucosal fibrosis score of 2 had lower relative serum sodium concentrations compared with cats with a score of 0 (adjusted *P* = .02; Figure 3A) and lower serum total calcium concentrations compared with cats with a score of 1 and 0 (adjusted *P* = .02 and adjusted *P* = .01, respectively; Figure 3B). No other significant associations were found between any of the remaining electrolyte variables and duodenal mucosal fibrosis scores (*P* > .07).

Cats with CIE and a colonic mucosal fibrosis score of 1 had higher absolute serum potassium concentrations compared with cats with a score of 0 (adjusted *P* = .03; Figure 3C) and lower sodium: potassium ratios compared with cats with a score of 0 (adjusted *P* = .01; Figure 3D). No other significant associations were found between the remaining electrolyte variables and colonic mucosal fibrosis scores (*P* > .06).

#### 3.3.4 Serum electrolytes and treatment subclassification

No significant associations were identified between any electrolyte variables and subclassification of cats with CIE based on response to treatment (food‐responsive, antibiotic‐responsive or immunosuppressant‐responsive; *P* > .08).

#### 3.3.5 Serum electrolytes and outcome

**FIGURE 4:**
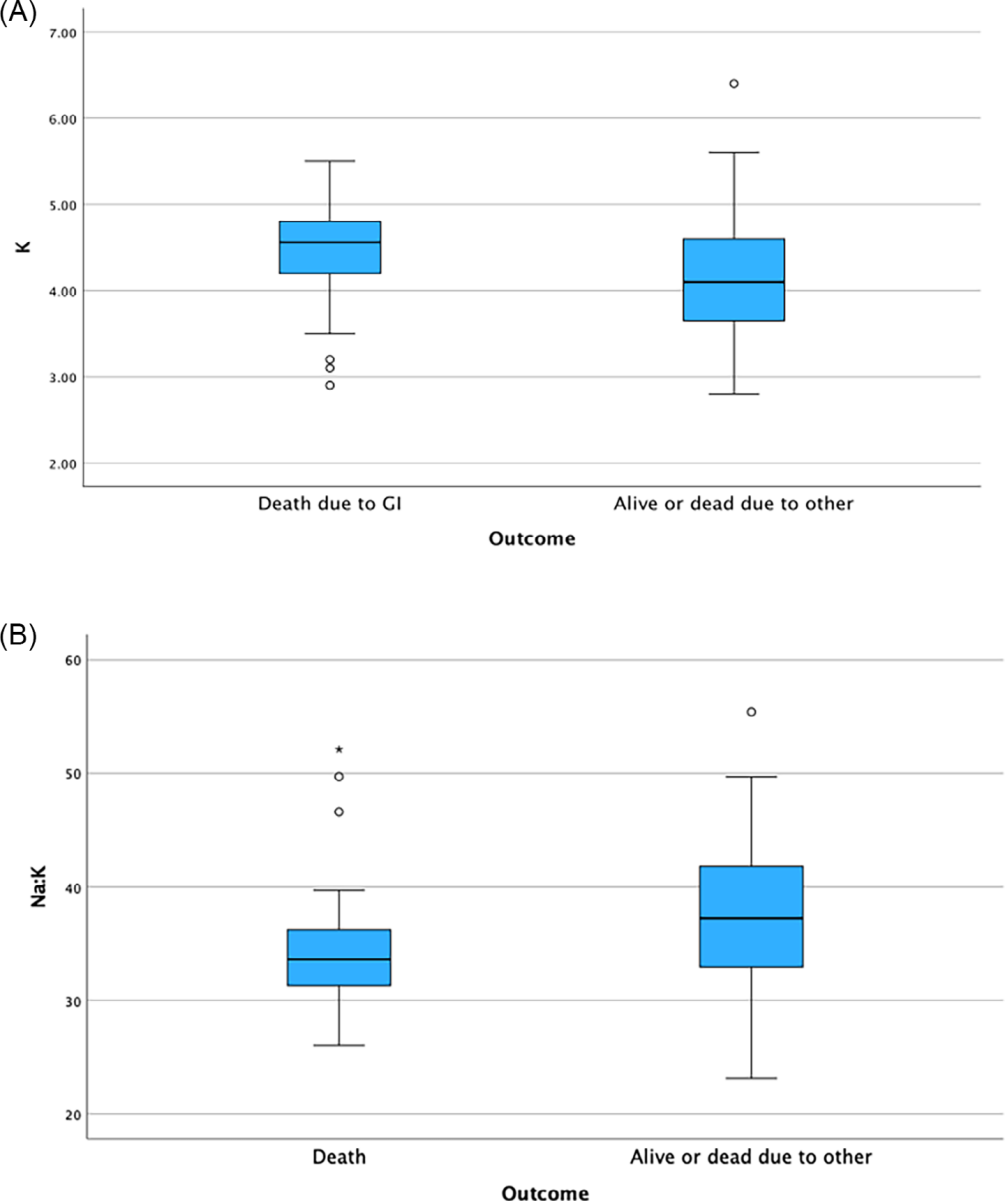
Box and whisker plot of (A) absolute serum potassium concentrations and (B) serum sodium: Potassium ratio in cats with chronic inflammatory enteropathy (CIE) that were either dead due to gastrointestinal (GI) disease or alive or dead due to other conditions. The box represents the interquartile range, with the bottom and top edges of the box representing the 25th and 75th percentiles, respectively. The vertical line that splits the box in 2 represents the median. The bottom and top whiskers represent the minimum and maximum values, unless there is an outlier. If an outlier is present (represented as a circle or a star if an extreme outlier), the whiskers represent 1.5 times the interquartile range. Cats that died as a result of their gastrointestinal disease had higher absolute serum potassium concentrations and lower sodium: Potassium compared to those cats that died of other causes or were alive at follow‐up (*P* = .02 and *P* = .01, respectively).

Cats that died or were euthanized as a result of their gastrointestinal disease had significantly higher absolute and relative serum potassium concentrations and lower sodium: potassium ratios compared with cats that died of other causes or were alive at follow‐up (*P* = .02, *P* = .02 and *P* = .01, respectively; Figure 4A,B). No other significant associations were found between the remaining electrolyte variables and outcome (*P* > .1).

## 4 DISCUSSION

Our results showed that, similar to both humans with IBD and dogs with CIE, cats with CIE also commonly have serum electrolyte abnormalities. In our study, hyponatremia and hypokalemia were documented in cats with CIE, which is similar to reports in humans with IBD and dogs with CIE.^9^, ^11^ The luminal epithelial surface of the colon is responsible for electrolyte transport.^15^ Therefore, in addition to its motor function, the colon is involved in both the absorption and secretion of electrolytes and water from the mucosal surface to the vascular side and also in the opposite direction.^16^ Colonic sodium absorption occurs primarily via electroneutral Na^+^/H^+^ and Cl^−^/HCO3^−^ exchange and via electrogenic epithelial sodium channels (ENaC), whereas potassium secretion is also largely maintained by the ENaC.^9^ In active ulcerative colitis in humans, the intestinal mucosa has lost this ability to absorb sodium, and therefore, a net decrease in sodium absorption occurs, which also results in impaired colonic water absorption.^17^ In addition, increased plasma‐to‐lumen sodium flux occurs, which suggests that the mucosa possesses increased leakiness.^18^ In ulcerative colitis patients, potassium loss into the colonic lumen is relatively excessive.^17^ Indeed, intestinal mucus from ulcerative colitis patients was shown to have high sodium and potassium content.^17^ In addition, the colonic mucosal cells in ulcerative colitis also were shown to have increased leakiness to potassium.^19^ Similar to ulcerative colitis, humans with Crohn's disease have lower serum sodium and potassium concentrations with similar mechanisms of decreased sodium absorption and increased potassium secretion.^20^ The mechanism of decreased sodium absorption and increased potassium secretion in ulcerative colitis and Crohn's disease is mainly attributed to the down‐regulation of ENaC.^21^ The aldosterone‐regulated ENaC is central to colonic and renal recovery of sodium and increased potassium secretion.^22^ One study showed that exposure of the distal colon of rats to the pro‐inflammatory cytokine TNF‐alpha resulted in decreased electrogenic sodium absorption because of impaired transcriptional gamma‐ENaC induction, which was similar to changes that were documented to occur in humans with Crohn's disease.^23^ In Na^+^/H^+^ exchanger 3 (NHE3) knock‐out mice, studies have shown that ENaC expression and function significantly increase in conditions associated with electrolyte loss and diarrhea.^24^ However, this compensatory function has been shown to be perturbed in ulcerative colitis patients, because of pro‐inflammatory cytokines preventing transcriptional induction of gamma‐ENaC, resulting in impaired sodium absorption and osmotic diarrhea.^25^, ^26^ Interestingly, dogs with CIE have been shown to upregulate ENaC at the level of the ileum, likely as a compensatory mechanism to increase intestinal sodium absorption.^27^ However, despite this upregulation in the ileum, an opposite effect was seen in the colon^28^ and 14% of dogs with CIE were documented to be hyponatremic.^11^ Furthermore, serum sodium concentration was associated with the severity of diarrhea and duodenal histopathology lesions in dogs with CIE.^11^ Therefore, it is likely that dysregulation of intestinal electrolyte transporters and serum sodium concentrations play roles in the pathophysiology or are a consequence of CIE in dogs, and additional studies should help to elucidate if these changes are caused by a loss of function of ENaC, or decreased ENaC expression. Similarly, additional studies are needed in cats with CIE to determine if the pathomechanism of hyponatremia is failure to upregulate or loss of function of ENaC.

In our study, hypokalemia was associated with vomiting in cats with CIE, which also was reported in dogs with CIE and vomiting.^11^ Hypokalemia was more frequent in dogs with CIE, affecting 19% of dogs compared with 10.6% of cats in our study, but only 61% of cats presented with vomiting in our study vs 79% in the CIE study in dogs.^11^ Also, additional factors such as increased severity or frequency of vomiting could have contributed to more dogs with CIE presenting with hypokalemia compared with the cats in our study. Alternatively, dogs with CIE may have higher colonic potassium secretion compared with cats with CIE, which could have contributed to the higher percentage of hypokalemia in this species. Similar to cats with CIE, the sodium: potassium ratio was significantly higher in CIE compared with healthy dogs.^11^ These similarities in electrolyte abnormalities between dogs and cats with CIE suggest a shared pathomechanism between the 2 species.

In our study, cats with CIE also presented with total hypocalcemia. The absorption of calcium occurs almost exclusively within the duodenum, jejunum, and ileum via the paracellular pathway, which is passive absorption based on factors such as dietary calcium intake and the transcellular pathway, an active process influenced by calcitriol.^29^ Total hypocalcemia in cats with CIE could be attributed to decreased intestinal absorption, decreased intake and vitamin D deficiency because of decreased absorption. Indeed, decreased vitamin D concentrations have been documented in cats with chronic enteropathy.^30^ Unfortunately, because vitamin D concentrations were unavailable for the cats in our study, we could not verify the pathomechanism for total hypocalcemia. Interestingly, in our study, serum total calcium concentrations, as well as sodium concentrations, were lower in cats with higher duodenal mucosal fibrosis scores. This finding suggests that intestinal fibrosis may impair the absorption, either passive or active, of these electrolytes. In the colon, higher fibrosis scores were associated with higher serum potassium concentrations and lower sodium: potassium ratios. In the colon, angiotensin (Ang) 2 has been shown to increase sodium and water reabsorption in rats via sodium chloride coupled transport.^28^ Angiotensin‐converting enzyme 2 (ACE2) catalyzes the conversion of Ang to Ang 1‐7, which negatively regulates the renin‐angiotensin system.^31^ Some studies have shown protective effects of ACE2, whereas others have shown that pharmacologic blockade of ACE2 attenuates inflammation in dextran sodium sulfate mice models.^32^, ^33^ Therefore, ACE2 may exert a dual protective or deleterious role within the gastrointestinal tract. Interestingly, ACE2 is increased in humans with IBD and evidence from both experimental and clinical studies of ACE2 activity indicate a role in the pathophysiology of this disease.^31^, ^34^, ^35^, ^36^, ^37^ Although the intestinal expression of ACE2 is inversely correlated with fibrosis, ACE was positively correlated with fibrosis in humans with IBD.^31^ Furthermore, components of both the traditional and alternative renin‐angiotensin system pathways were increased in dogs with CIE compared with healthy controls.^27^ Therefore, given the role of ACE2 and the renin‐angiotensin system in intestinal inflammation, fibrosis and electrolyte transport, these pathways could explain the association of duodenal and colonic fibrosis and serum electrolyte concentrations seen in cats with CIE in our study. Additional studies should help determine if the expression or function of intestinal transmembrane ACE2, other components of the traditional or alternative arm of the renin‐angiotensin system or both, and electrolyte transporters, including ENaC, are impaired in cats with CIE, and whether such effects could explain the association of fibrosis with some of the electrolyte imbalances seen in cats with CIE in our study.

Our study showed that cats with CIE that died because of their gastrointestinal disease had higher serum potassium concentrations and lower sodium: potassium ratios than cats that were alive or died or were euthanized for other causes. These electrolyte imbalances may represent more severe gastrointestinal disease, which may result in decreased potassium secretion and less sodium absorption in the colon. Alternatively, because these same electrolyte imbalances were associated with colonic fibrosis, and colonic fibrosis previously has been associated with death as a result of gastrointestinal disease in cats with CIE,^12^ these electrolyte changes could be acting as confounders representing colinear variables because of their association with colonic fibrosis. Additional studies may help determine if serum electrolyte imbalances independently affect outcome in cats with CIE, or if any abnormalities are a result of their associations with colonic fibrosis. If the latter is identified, then serum electrolyte concentrations may act as a surrogate marker for the presence or severity of colonic fibrosis and eventual outcome in these cats.

Our study also showed that cats with acute GE had significantly lower serum sodium concentrations compared with healthy control cats. This finding is interesting because it could suggest serum sodium concentrations decrease at the onset of gastrointestinal disease and therefore may not be related to chronicity. However, because cats with acute GE were significantly younger than cats in the other groups, with a median age of 5 months, younger age may have impacted their serum sodium concentrations.^38^ Interestingly, a higher proportion of cats with acute GE presented with serum phosphorus concentrations above the reference interval compared with the other 3 groups. This finding could indicate a pre‐renal cause, such as dehydration or hypovolemia in cats with acute GE or again could signify the younger age of these cats and therefore their growth.^39^ Future studies utilizing an age‐matched acute GE group should help ascertain whether the changes seen in acute GE in our study were a consequence of the disease itself or age. Furthermore, although our study found significant differences in electrolyte concentrations among the 4 groups of cats, and associations with certain clinical signs, intestinal mucosal fibrosis scores, and outcome in CIE, additional studies are needed to determine the clinical relevance of these findings.

Finally, in our study, hyperchloremia was the most frequent electrolyte abnormality in cats with CIE, affecting 26 (19.7%) cats. This finding is interesting because it differs from what occurs in humans with IBD, in whom hypochloremia is documented.^9^ Hyperchloremia has several possible causes including artifacts (eg, lipemia), iatrogenic (eg, administration of chloride‐containing fluids such as hypertonic saline), and hyperchloremic metabolic acidosis.^40^, ^41^ The former 2 causes were deemed unlikely in our study, because owners are instructed to fast their cats before appointments at both referral hospitals and serum electrolyte concentrations were measured at presentation, before any treatment. Therefore, the likely cause of hyperchloremia in cats with CIE in our study could be hyperchloremic metabolic acidosis of which 2 main causes exist.^40^, ^41^ The first is bicarbonate loss either from primary causes, such as gastrointestinal loss via vomiting, secretory diarrhea, or renal causes such as proximal renal tubular acidosis or secondary causes including compensatory response to a primary respiratory alkalosis, such as hyperventilation or hypocapnia from pulmonary disease or pain. However, because no association was found between clinical signs, such as diarrhea and vomiting, and serum chloride concentration in cats with CIE in our study, gastrointestinal loss may have been less likely as a cause of the hyperchloremia. The second cause of hyperchloremia is bicarbonate consumption, either associated with the production of a noncarbonic acid, such as lactate, or excretion of a noncarbonic acid with renal disease. Therefore, the reason for hyperchloremia in cats in our study requires further investigation of blood pH, bicarbonate concentrations and base excess in these cats. Unfortunately, parameters related to acid‐base status were not available for all cats in our study and therefore could not be assessed further. Although acid‐base disturbances have been reported to occur in humans with IBD, mild to severe metabolic alkalosis is seen typically.^9^ However, dogs with chronic enteropathy have been shown to have increased fecal lactate concentrations,^42^ which could be associated with metabolic acidosis, and therefore, additional studies to determine the cause of hyperchloremia in cats with CIE should focus on this metabolite.

Limitations of our study include its retrospective design and reliance on hospital records, which were not always complete or were missing relevant information, such as the feline chronic enteropathy activity index,^43^ precluding use of this variable in statistical analyses. Other limitations included the use of total calcium and magnesium concentrations rather than ionized concentrations. Additionally, 1 of the laboratories does not have a total magnesium concentration reference interval and, therefore, the proportion of cats with concentrations below, within or above a reference interval for this variable could not be determined. Because our study was retrospective, gastrointestinal biopsy specimens were already collected at the time of diagnosis, which did not allow for broader assessment of the gastrointestinal tract. Therefore, neoplasia may have been missed in other intestinal segments.^2^ Similarly, an underlying CIE cannot be ruled out in all cats categorized as acute GE, as well as intestinal disease in blood donor cats, which also could have caused an overlap in disease categories. Similarly, cats in the different treatment subcategories could have overlapped, for example, if dietary therapy was not tried or exhausted before immunosuppressive treatment. Finally, because our study occurred at 2 centers, >1 pathologist evaluated histopathology for the cats with CIE. Therefore, future studies should evaluate all scores by a single pathologist to minimize inter‐pathologist variation.

In conclusion, we identified serum electrolyte abnormalities in cats with CIE, with the most common being hyperchloremia, followed by hypokalemia, hyponatremia, and hypocalcemia. Cats with CIE with higher duodenal mucosal fibrosis scores had lower serum sodium and total calcium concentrations and those cats with higher colonic mucosal fibrosis scores had higher serum potassium concentrations and lower sodium: potassium ratios. Finally, in cats with CIE, higher serum potassium concentrations and lower sodium: potassium ratios were associated with a higher likelihood of death caused by gastrointestinal disease. Additional studies are needed to determine the clinical relevance of these findings and whether or not these electrolyte abnormalities in cats with CIE could be caused by failure to upregulate ENaC or loss of function of ENaC, induction of intestinal transmembrane ACE2, or dysregulated expression or function of the renin‐angiotensin pathway, or other intestinal electrolyte transporters.

## CONFLICT OF INTEREST DECLARATION

Authors declare no conflict of interest.

## OFF‐LABEL ANTIMICROBIAL DECLARATION

Authors declare no off‐label use of antimicrobials.

## INSTITUTIONAL ANIMAL CARE AND USE COMMITTEE (IACUC) OR OTHER APPROVAL DECLARATION

Approved by the Royal Veterinary College (URN SR2024‐0045R). Approval was not required by the University of Leipzig.

## HUMAN ETHICS APPROVAL DECLARATION

Authors declare human ethics approval was not needed for this study.
